# Research on electroacupuncture parameters for knee osteoarthritis based on data mining

**DOI:** 10.1186/s40001-022-00795-9

**Published:** 2022-08-31

**Authors:** Fei-hong Cai, Fan-lian Li, Yu-chen Zhang, Pei-qi Li, Bin Xiao

**Affiliations:** College of Acupuncture and Tuina, Shanghai University of TCM, Shanghai, 201203 China

**Keywords:** Electroacupuncture, Knee osteoarthritis, Parameter, Data mining

## Abstract

**Background:**

Knee osteoarthritis, a common degenerative joint disease, has been widely treated by electroacupuncture in recent years. However, there are too many parameters of the treatment currently, resulting in various applications in clinical practice. This study aims to summarize the optimal stimulation parameters of electroacupuncture for knee osteoarthritis in clinical studies by applying data mining techniques.

**Methods:**

Four databases including Pubmed, Cochrane Library, Embase, and Web of Science were searched for clinical studies on electroacupuncture treating knee osteoarthritis from 2012 to 2021. A database was established by Microsoft Excel 2020 and analyzed by R Version 4.1.1.

**Results:**

Forty-six articles were included according to the established criteria. The most used electroacupuncture stimulation parameters were 0.30 mm × 40 mm needle, continuous wave, low frequency of current (mainly 2 Hz), stimulation duration for 30 min per treatment, and frequency of treatment for once a day. Eighteen acupoints were mentioned and the most used ones include Dubi (ST35), Liangqiu (ST34), Neixiyan (EX-LE4), Xuehai (SP10), Yanglingquan (GB34), and Yinlingquan (SP9), and those most generally used acupoints are closely arranged on the Stomach Channel of Foot Yangming. Cluster analysis showed two groups, one for obligatory acupoints and one for adjunctive ones. The association analysis showed the most supported acupoint pair was Liangqiu (ST34) and Xuehai (SP10).

**Conclusions:**

Continuous wave, low frequency of current (2 Hz), 30-min stimulation, and local acupoint selection are frequently used for electroacupuncture treating knee osteoarthritis. Due to the limitations of this study, further research and more standardized, multi-centered, and large-sample clinical trials should be conducted to provide more convincing evidence.

## Background

Knee osteoarthritis (KOA) is a common degenerative joint disease worldwide, especially for the aged [1, 2]. According to the U.S. National Health Interview Survey [3], there were about 14 million people diagnosed with symptomatic KOA in the U.S. In a data analysis conducted by China Health and Retirement Longitudinal Study (CHARLS), a 4-year national census in China, the cumulative incidence of symptomatic KOA was 8.5% in 4 years among Chinese adults over 45 years old. It is estimated that the elder population with symptomatic KOA in China has amounted to 37.35 million. China is the most populous country in the world, and along with the aging of population, the incidence of KOA will accordingly rise [4].

The typical symptoms and signs of KOA include persistent knee pain, joint swelling and deformity, joint stiffness, numbness, limited movement, and even disability for further development [5, 6], which can greatly interfere with people’s quality of life. Unfortunately, there are still no specific drugs for KOA [7]. The traditional treatment is drug intervention, such as analgesics and non-steroidal anti-inflammatory drugs (NSAIDs), mainly for relieving symptoms and improving quality of life [8]. Nevertheless, medications of long-term courses not only increase patients’ financial burden [9, 10], but also cause uncertain adverse reactions [11] including bleeding, gastric perforation, and increased risk of cardiovascular and renal diseases [12, 13]. In recent years, the treatment of KOA has gradually shifted from drug intervention to non-drug one [5].

Acupuncture, a non-drug treatment in traditional Chinese medicine (TCM), has been proved effective in analgesia for chronic pain [14–17]. In TCM theory, KOA refers to “Bi Syndrome” (arthralgia syndrome), a disease in the tendons and bones of the knee. The major etiology and pathogenesis are the insufficiency of liver and kidney, and the external invasion of wind, cold, and dampness pathogens [18, 19]. Acupuncture treats KOA from two respects: unblocking the channels and pain relief. Meta-analysis showed that acupuncture has good efficacy in relieving KOA pain and improving physical function in the short term, with a lower risk of adverse effects [1, 20]. Electroacupuncture (EA) is a modified method of traditional acupuncture, combining needle stimulation and electrical stimulation. It not only expands the therapeutic range of traditional acupuncture, but also promotes the efficacy by relieving muscle spasms, thus exerting effects such as eliminating inflammation, improving blood circulation, and relieving pain [21–23]. Studies [24, 25] have proven that electroacupuncture can relieve KOA symptoms of pain, swelling, stiffness, and inflexible movement. Fu et al. [26] applied three methods (electroacupuncture, acupuncture, and western medicine) to treat KOA, and found that they had comparable effects in the short term, while electroacupuncture had significantly better efficacy than the other two in the long term, with a longer duration as well.

Electroacupuncture has the characteristic of parameter objectification. When it is used for treatment, the selection and application of parameters play essential roles in satisfactory efficacy [27]. The selection and quantification of electroacupuncture stimulation parameters have come to center stage in the research on the clinical practice and principles of acupuncture [28]. However, there are too many parameters currently, resulting in various applications in clinical practice [29]. Compared with the popularity of electroacupuncture treating KOA, there is a lack of research on specific electroacupuncture parameters for KOA [30]. Therefore, this study focused on literature studying electroacupuncture treating KOA in the recent decade. Data mining techniques were used to extract and analyze different electroacupuncture parameters from the articles, such as waves, frequency of current, duration, and acupoints. We summarized the application rules of electroacupuncture parameters in the treatment of KOA, hoping to provide a reference for EA standardization and clinical practice.

## Methods

### Retrieval strategy

We searched Pubmed, Cochrane Library, Embase, Web of Science databases from January 2011 to December 2021. Search keywords were mainly used as follows and modified according to different databases: *“electro-acupuncture”*, *“electroacupuncture”*, *“electrical acupuncture”*, *“EA”*, *“Osteoarthritis, Knee”*, *“Knee Osteoarthritis”*, *“Osteoarthritis of Knee”*, *“KOA”*. More relevant studies were manually searched. There was no language limitation.

### Inclusion criteria

1. Randomized controlled trials regarding patients clearly diagnosed with knee osteoarthritis.
2. Studies that apply electroacupuncture as the major intervention or with additive treatments.
3. Clinical trials on electroacupuncture treatment with explicit acupoint selection.
4. Studies with the same data and results were regarded as one article.

### Exclusion criteria

1. Nonclinical studies such as animal experiment, review, meta-analysis, protocol, erratum, etc.
2. Studies that do not apply electroacupuncture as the major intervention.
3. Studies on participants having KOA symptoms, but not clearly diagnosed.
4. Studies that do not list any of the parameters observed in this study, or only describe the general information, without any detail.

### Literature quality management

Three levels of quality control were strictly used for literature management. Firstly, all the titles and abstracts of the retrieved articles were screened by two independent reviewers (YC Zhang and PQ Li), and those which did not meet the criteria were excluded. The second screening was performed by reading the remaining articles in full text, mainly for the clearly stated parameters (frequency of current, wave, stimulation duration, etc.) and the statistical significance of the difference in efficacy between the treatment and control groups. Finally, another two researchers (FH Cai and FL Li) were responsible for the complete examination and extracted data with a predefined form. Any disagreement in the process was resolved by discussion between the two reviewers or consultation with a third reviewer (B Xiao).

### Data extraction

Microsoft Excel 2020 was used to establish the database. Eleven items including title, first author’s name, year of publication, needle type, frequency of treatment, courses of treatment, stimulation duration, frequency of current, wave, pairs of acupoints connected to electrodes, and acupoints selection are recorded in the database.

### Data analysis

R Version 4.1.1 was used to analyze the data. Frequency analysis was performed to count target items. The Apriori algorithm was used to show the association among electroacupuncture stimulation parameters and acupoints. Ward’s method was used to cluster the selected acupoints.

## Results

### Retrieval of studies

**Fig. 1:**
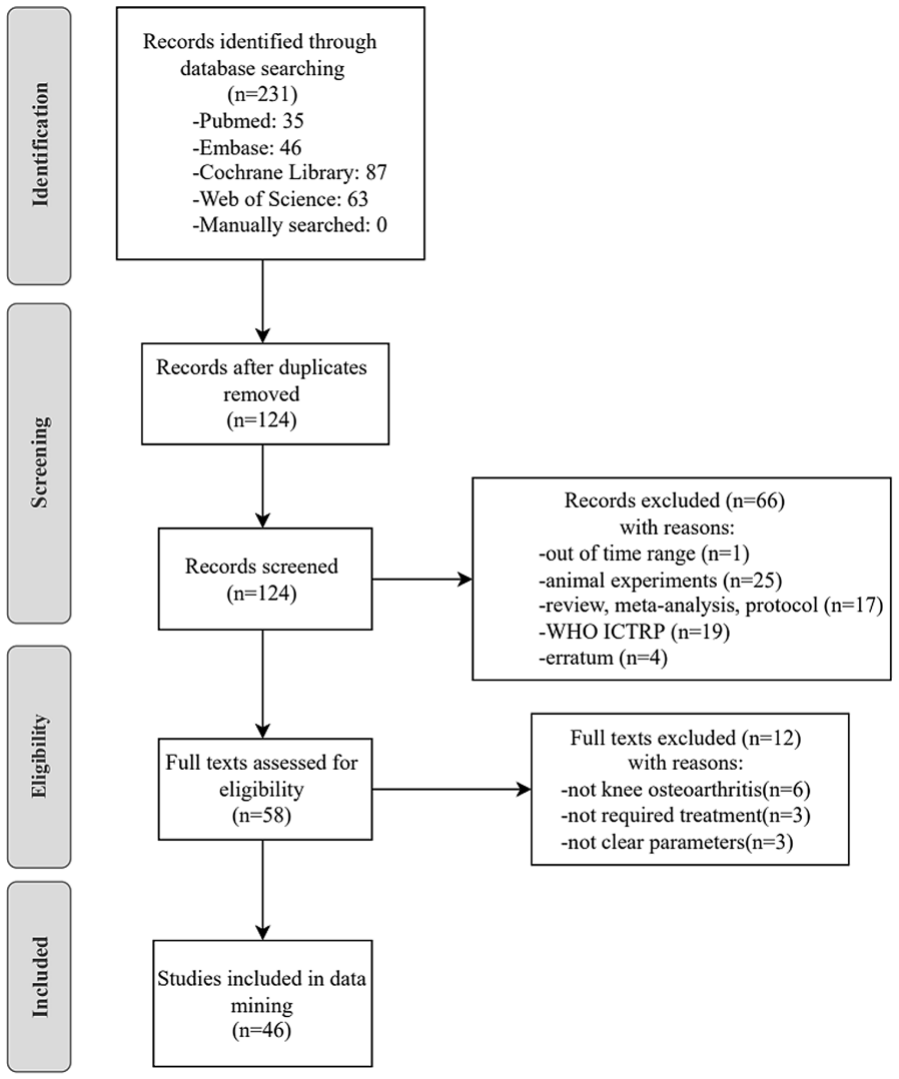
Flow diagram for selection of articles

A total of 231 articles were found in the four databases and then imported into Endnote Version 20.2.0 for selection. Among these, 107 were removed for duplicates. Then 66 articles were excluded by screening the titles and abstracts; 12 articles were eliminated through full-text assessment. 46 articles were finally included for further analysis. The exclusion reasons and selection flow are shown in Fig. 1.

### Frequency of needle type

Ten types of needles were mentioned in 38 studies. The two most used needle types were: 0.30 mm × 40 mm and 0.25 mm × 40 mm, as shown in Table 1.

**Table 1:** Frequency of needle type

Number	Needle type (mm)	Frequency (%)
1	0.30 × 40	19 (41.30)
2	0.25 × 40	11 (23.91)
3	0.25 × 30	4 (8.70)
4	0.30 × 50	3 (6.52)
5	0.25 × 25	3 (6.52)
6	0.32 × 50	2 (4.35)
7	0.30 × 25	1 (2.17)
8	0.30 × 30	1 (2.17)
9	0.30 × 75	1 (2.17)
10	0.32 × 40	1 (2.17)

### Frequency of treatment

Eight kinds of frequency of treatment were mentioned in 43 studies. The top three were: once a day (30.23%), three times per week (20.93%), once every other day (18.60%), as shown in Table 2.

**Table 2:** Frequency of treatment

Number	Frequency of treatment	Frequency (%)
1	Once a day	13 (30.23)
2	Three times per week	9 (20.93)
3	Once every other day	8 (18.60)
4	Five times per week	4 (9.30)
5	Four times per week	4 (9.30)
6	Twice a week	3 (6.98)
7	Once a week	1 (2.33)
8	One and a half times per week	1 (2.33)

### Frequency of electroacupuncture parameters

#### Frequency of waveform

Thirty-six studies mentioned the waveforms. Continuous wave (63.89%) was used significantly more frequently than dilatational wave (36.11%), as shown in Table 3.

**Table 3:** Frequency of waveform

Number	Waveform	Frequency (%)
1	Continuous wave	23 (63.89)
2	Dilatational wave	13 (36.11)

#### Frequency of current

Fourteen kinds of frequency of current were mentioned in forty studies, ranging from 2 to 100 Hz. The top three were: 2 Hz (37.50%), 2/100 Hz (25.00%), 20 Hz (10.00%), as shown in Table 4.

**Table 4:** Frequency of current

Number	Frequency of current (Hz)	Frequency (%)
1	2	15 (37.50)
2	2/100	10 (25.00)
3	20	4 (10.00)
4	4	1 (2.50)
5	5	1 (2.50)
6	10	1 (2.50)
7	100	1 (2.50)
8	10–30	1 (2.50)
9	2/10	1 (2.50)
10	2/15	1 (2.50)
11	2–4	1 (2.50)
12	3/100	1 (2.50)
13	50–80	1 (2.50)
14	60–80	1 (2.50)

#### Frequency of stimulation duration

Three kinds of stimulation duration were mentioned in 46 studies, including 30 min (76.09%), 20 min (19.57%), and 25 min (6.52%), as shown in Table 5.

**Table 5:** Frequency of stimulation duration

Number	Stimulation duration (min)	Frequency (%)
1	30	35 (76.09)
2	20	8 (17.40)
3	25	3 (6.52)

### Association analysis of electroacupuncture parameters

**Fig. 2:**
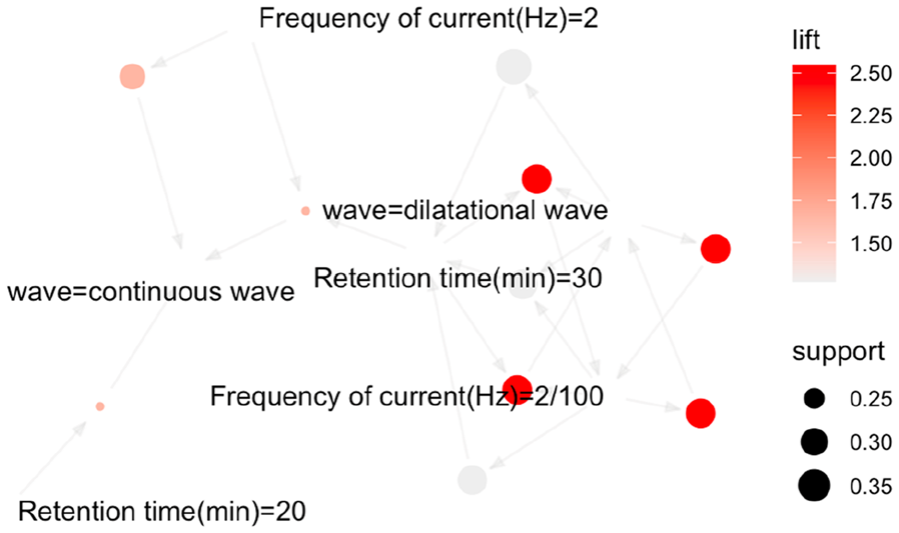
Network diagram of association rules of electroacupuncture parameters. *The nodes in the figure are the left-hand side (LHS) and right-hand side (RHS) of the association rule, and the arrow direction is from LHS to RHS. The width of the arrow indicates the degree of support, and the grayscale indicates the level of lift. “Retention time” refers to stimulation duration

The association analysis was performed on three of the parameters (waveform, frequency of current, stimulation duration), with the setting of support degree (0.3) and confidence degree (0.8). A total of ten association rules were obtained by Apriori algorithm, as shown in Table 6. The visualization result is shown in Fig. 2. The results indicated that a combination of dilatational wave, 30-min stimulation, and current of 2/100 Hz was most applied for electroacupuncture treating KOA.

**Table 6:** Association analysis of electroacupuncture parameters

Number	Association rules	Support	Confidence	Coverage	Lift	Count
1	[Retention time (min) = 20]→[wave = continuous wave]	0.21	1	0.21	1.65	6
2	[Frequency of current (Hz) = 2]→[wave = continuous wave]	0.29	1	0.29	1.65	8
3	[Frequency of current (Hz) = 2/100]→[wave = dilatational wave]	0.32	1	0.32	2.55	9
4	[Wave = dilatational wave]→[Frequency of current (Hz) = 2/100]	0.32	0.82	0.39	2.55	9
5	[Frequency of current (Hz) = 2/100]→[Retention time (min) = 30]	0.32	1	0.32	1.27	9
6	[Wave = dilatational wave]→[Retention time (min) = 30]	0.39	1	0.39	1.27	11
7	[Retention time (min) = 30, Frequency of current (Hz) = 2]→[wave = continuous wave]	0.21	1	0.21	1.65	6
8	[Frequency of current (Hz) = 2/100, wave = dilatational wave]→[Retention time (min) = 30]	0.32	1	0.32	1.27	9
9	[Retention time (min) = 30, Frequency of current (Hz) = 2/100]→[wave = dilatational wave]	0.32	1	0.32	2.55	9
10	[Retention time (min) = 30, wave = dilatational wave]→[Frequency of current (Hz) = 2/100]	0.32	0.82	0.39	2.55	9

### Analysis of acupoints and channels

#### Analysis of acupoints

Eighteen acupoints were mentioned in the studies included. The total frequency of acupoints involved was 157 times, and each acupoint was used 8.72 times on average. The top five acupoints with the highest frequency were Dubi (ST35), Liangqiu (ST34), Neixiyan (EX-LE4), Xuehai (SP10), and Yanglingquan (GB34), with the cumulative frequency accounting for 63.06% of the total. Seven acupoints were used more frequently than the mean number, with a cumulative frequency accounting for 80.26% of the total, as shown in Table 7.

**Table 7:** Frequency of acupoints

Number	Acupoint	Frequency (%)
1	Dubi (ST35)	22 (14.01)
2	Liangqiu (ST34)	21 (13.38)
3	Neixiyan (EX-LE4)	21 (13.38)
4	Xuehai (SP10)	18 (11.46)
5	Yanglingquan (GB34)	17 (10.83)
6	Yinlingquan (SP9)	14 (8.92)
7	Zusanli (ST36)	13 (8.28)
8	Xiyan (EX-LE5)	6 (3.82)
9	Ashi point	5 (3.18)
10	Heding (EX-LE2)	5 (3.18)
11	Xiyangguan (GB33)	5 (3.18)
12	Ququan (LR8)	4 (2.55)
13	Dachangshu (BL25)	1 (0.64)
14	Qihaishu (BL24)	1 (0.64)
15	Shenshu (BL23)	1 (0.64)
16	Weizhong (BL40)	1 (0.64)
17	Xuanzhong (GB39)	1 (0.64)
18	Yinbao (LR9)	1 (0.64)

#### Analysis of channels

The eighteen acupoints were statistically analyzed for the channels they belong to. Six channels were involved, of which the Stomach Channel of Foot Yangming was most used. The top three with the highest frequency were the Stomach Channel of Foot Yangming, the Spleen Channel of Foot Taiyin, and extra points (non-channel points), with the cumulative frequency accounting for 76.43% of the total. Frequency of acupoints and of the channels they belong to are shown in Table 8.

**Table 8:** Frequency of channels

Number	Channel	Count of acupoints	Frequency (%)
1	The stomach channel of foot Yangming	3	56 (35.67)
2	The spleen channel of foot Taiyin	2	32 (20.38)
3	Extra point (non-channel point)	3	32 (20.38)
4	The gallbladder channel of foot Shaoyang	3	23 (14.6)
5	The liver channel of foot Jueying	2	5 (3.2)
6	Ashi point (ouch point)	1	5 (3.2)
7	The bladder channel of foot Taiyang	4	4 (2.5)

Frequency ratio = cumulative frequency of acupoints belonging to the same channel/total frequency of all the acupoints involved

### Association analysis of acupoints

**Fig. 3:**
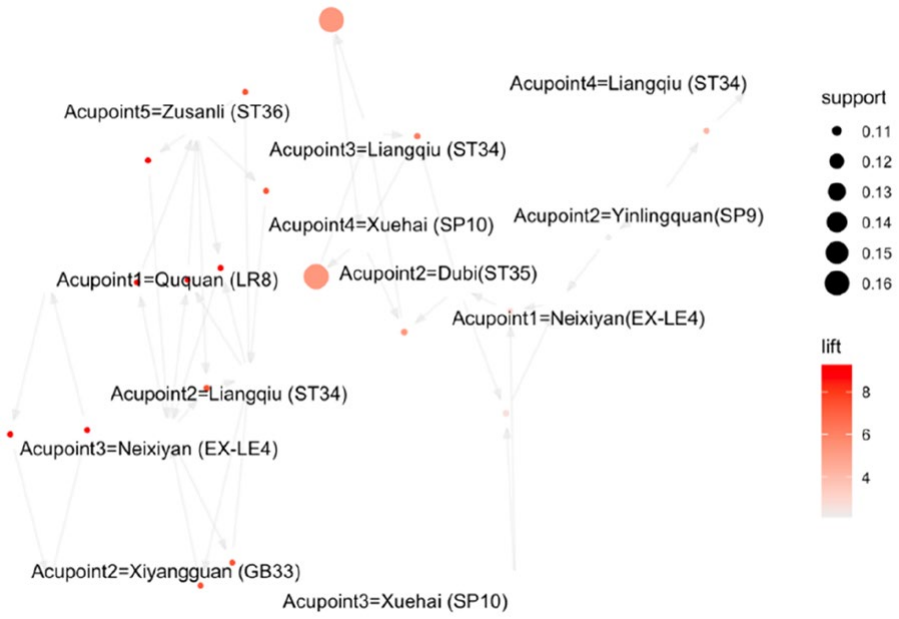
Network diagram of association rules of acupoints. *The nodes in the figure are the left-hand side (LHS) and right-hand side (RHS) of the association rule, and the arrow direction is from LHS to RHS. The width of the arrow indicates the degree of support, and the grayscale indicates the level of lift

The association analysis was performed on acupoints used at least 4 times, with the setting of support degree (0.1) and confidence degree (0.5). A total of 19 association rules were obtained by Apriori algorithm, as shown in Table 9. The visualization result is shown in Fig. 3. The results indicated that Liangqiu (ST34) and Xuehai (SP10) had a stronger association.

**Table 9:** Association analysis of acupoints

Number	Association rule	Support	Confidence	Coverage	Lift	Count
1	[Ququan (LR8)]→[Xiyangguan (GB33)]	0.11	1.00	0.11	9.25	4
2	[Xiyangguan (GB33)]→[Ququan (LR8)]	0.11	1.00	0.11	9.25	4
3	[Yinlingquan (SP9)]→[Liangqiu (ST34)]	0.11	0.80	0.14	4.23	4
4	[Yinlingquan (SP9)]→[Neixiyan (EX-LE4)]	0.11	0.80	0.14	2.11	4
5	[Xuehai (SP10)]→[Liangqiu (ST34)]	0.16	1.00	0.16	5.29	6
6	[Liangqiu (ST34)]→[Xuehai (SP10)]	0.16	0.86	0.19	5.29	6
7	[Zusanli (ST36)]→[Neixiyan (EX-LE4)]	0.11	1.00	0.11	9.25	4
8	[Neixiyan (EX-LE4)]→[Zusanli (ST36)]	0.11	1.00	0.11	9.25	4
9	[Zusanli (ST36)]→[Liangqiu (ST34)]	0.11	1.00	0.11	7.40	4
10	[Liangqiu (ST34)]→[Zusanli (ST36)]	0.11	0.80	0.14	7.40	4
11	[Neixiyan (EX-LE4)]→[Liangqiu (ST34)]	0.11	1.00	0.11	7.40	4
12	[Liangqiu (ST34)]→[Neixiyan (EX-LE4)]	0.11	0.80	0.14	7.40	4
13	[Dubi(ST35), Xuehai (SP10)]→[Liangqiu (ST34)]	0.11	1.00	0.11	5.29	4
14	[Dubi(ST35), Liangqiu (ST34)]→[Xuehai (SP10)]	0.11	1.00	0.11	6.17	4
15	[Dubi(ST35), Xuehai (SP10)]→[Neixiyan(EX-LE4)]	0.11	1.00	0.11	2.64	4
16	[Neixiyan(EX-LE4), Xuehai (SP10)]→{Dubi(ST35)}	0.11	1.00	0.11	2.85	4
17	[Neixiyan (EX-LE4), Zusanli (ST36)]→[Liangqiu (ST34)]	0.11	1.00	0.11	7.40	4
18	[Liangqiu (ST34), Zusanli (ST36)]→[= Neixiyan (EX-LE4)]	0.11	1.00	0.11	9.25	4
19	[Liangqiu (ST34), Neixiyan (EX-LE4)]→[Zusanli (ST36)]	0.11	1.00	0.11	9.25	4

### Cluster analysis of acupoints

**Fig. 4:**
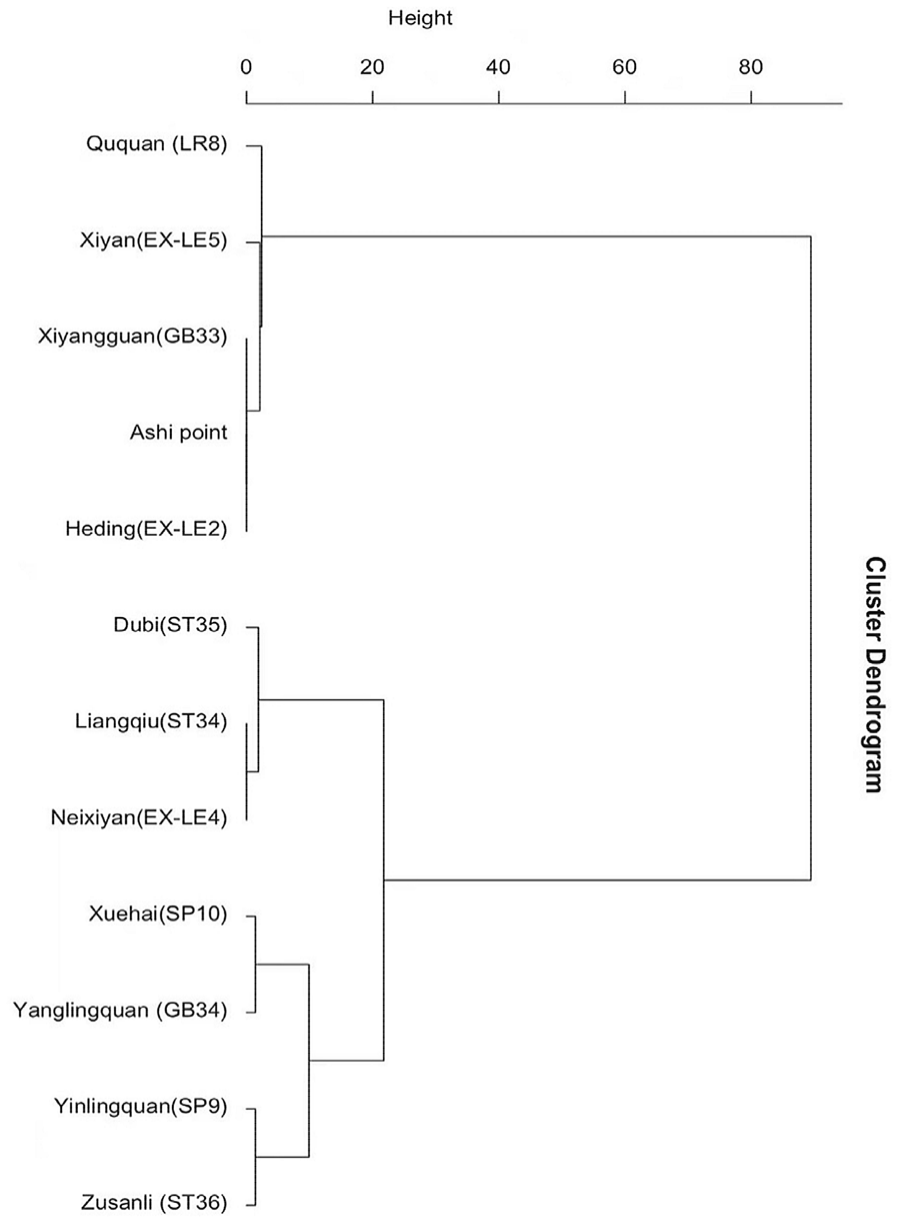
Cluster dendrogram of acupoints

A cluster analysis was performed on acupoints used at least 4 times. Two categories of effective clusters were obtained. One was 3 groups of acupoints: Dubi (ST35)–Liangqiu (ST34)–Neixiyan (EX-LE4), Xuehai (SP10)–Yanglingquan (GB34), and Yinlingquan (SP9)–Zusanli (ST36). The other was Ququan (LR8)–Xiyan (EX-LE5)–Xiyangguan (GB33)–Ashi point (Ouch point)–Heding (EX-LE2); see Fig. 4.

## Discussion

In clinical practice, the type of acupuncture needle varies due to several factors, including the patient’s constitution and the duration of the disease. This leads to difficulty in unifying the standards of needles, while different sizes can result in different effects [31]. The result of frequency analysis showed that in electroacupuncture treatment for KOA, the types of needles were used differently, with 0.30 mm × 40 mm most used. In terms of length, an acupuncture needle should be long enough to reach the proper depth within the range of the acupoint [32]. The frequency analysis of acupoints indicated that Dubi (ST35), Liangqiu (ST34), Neixiyan (EX-LE4), Xuehai (SP10), Yanglingquan (GB34), and Yinlingquan (SP9) were most used in treating KOA. According to *Channels–Collaterals and Acupoints* [33], the depth of needle insertion at these acupoints should be 1 cun to 1.5 cun (25 mm–40 mm), which a 40 mm needle can reach. Furthermore, several studies [25, 34, 35] have achieved satisfactory results by applying electroacupuncture to treat KOA with 40 mm needles and inserted at a depth of 25–40 mm. In *The Spiritual Pivot*, a TCM classic on acupuncture and acupoints, it says, “The key point of needling is that effect is achieved on the arrival of qi.” Electroacupuncture can improve the effect of needling through electrical stimulation based on the arrival of qi. Li et al. [36] studied the neurohumoral-immune regulatory networks in modern medicine and found that the relative equilibrium of yin-yang in TCM is similar to the networks maintaining the homeostasis of the body's internal environment. They also studied the human anatomy and found that the networks were most densely distributed in the dermis, and the deeper it went, the fewer networks it had. Therefore, it was concluded that the maximum amount of stimulation could be achieved by acupuncture to the sub-dermis and deeper needling was unnecessary. In terms of diameter, the literature study by Wang et al. [37] showed that 0.30 mm needles were most used, which is consistent with our study. Needles with larger diameters can stimulate receptors over a larger range and thus have a better effect. Nevertheless, the sensation will be more painful and less acceptable. Wang et al. [38] studied the effect of thick and thin filiform needles on the pain threshold of patients with sciatica and found that both could enhance the pain threshold, but thick ones had a more significant effect. Although thin needles cause lesser pain, they may affect the manipulation of needling, especially various techniques. Therefore, acupuncture needles of medium size are suggested for selection.

Frequency analysis of waveforms showed that continuous wave was most used for KOA. Huang et al. [39] found that continuous wave significantly improved symptoms such as knee pain, joint swelling, and instability. Continuous wave is a single disperse wave or dense wave. Dense wave can immediately relieve pain, while disperse wave maintains the analgesic effect. The consistent pulse width prevents discomfort in patients [40]. Furthermore, a literature review by Wang et al. [30] showed that continuous wave was the only choice for treatment in English articles, suggesting that the stimulation of the continuous wave at a fixed frequency might be easier to quantify than that of the dilatational wave. However, another research by Zhang et al. [41] proposed that continuous wave was more likely to cause electroacupuncture tolerance than the dilatational wave, and therefore reduced the efficacy. The dilatational wave can cause immediate and delayed inhibitory effects on sensory and motor nerves, coming up with better analgesic effect and longer duration. Besides, the dilatational wave is a variable stimulation that makes it harder for patients to adapt. Fang et al. [42] concluded that dilatational wave or continuous wave were the prior choices for electroacupuncture analgesia. Therefore, the optimal waveform of electroacupuncture treatment for knee osteoarthritis remains to be demonstrated by a large number of trials.

The frequency of current is an important parameter of electroacupuncture stimulation. Different frequencies can produce distinct reactions through various neurochemical mechanisms integrated by different central pathways, and also cause the release of different neurochemicals in the central nervous system (CNS), thus having different effects on the organisms [43, 44]. The accepted classification of frequencies of current is that 2 Hz to 5 Hz for low frequency, 15 Hz to 30 Hz for medium frequency, and 50 Hz to 100 Hz for high frequency. Our study showed that the frequency of current used in electroacupuncture treatment for KOA varied from 2 to 100 Hz, mainly for low frequency with 2 Hz most. The continuous wave frequency was dominated by 2 Hz, while the dilatational wave frequency was dominated by 2/100 Hz. It was found [45, 46] that 2 Hz electroacupuncture mainly activated the ENKergic system in the CNS and the β-EPergic system in the brain to mediate the analgesic effect, while 100 Hz electroacupuncture mainly activated the DYNergic system in the spinal cord to mediate the analgesic effect. Low-frequency electroacupuncture can suppress neuropathic pain more effectively than high-frequency one [20]. Han [47] proposed that dilatational wave with low frequency (2 Hz) and high frequency (15 Hz or 100 Hz) alternating for 3 s each resulted in the simultaneous release of three opioid peptides, producing stronger analgesic effects both in animal experiments and in clinical practice. A dilatational wave with 2 Hz and 100 Hz alternating is the ideal stimulation that induced the release of dynorphin (Dyn) and endorphin, while simultaneously stimulating two different acupoints with 2 Hz and 100 Hz current separately only induced the release of dynorphin [48]. The frequency of current for electroacupuncture treating KOA remains to be explored. Based on the current research, future studies can further compare the efficacy of 2/100 Hz and 2 Hz current for the treatment of KOA.

Our study indicated that the stimulation duration of electroacupuncture for KOA varied from 20 to 30 min, with 30 min used most. Wang et al. [30] found that the clinical electroacupuncture stimulation duration was mainly from 25 to 45 min and 30-min treatment was used most, which achieved good efficacy and was in accordance with the course of acupuncture action. Ye et al. [49] also reported that a 30-min electroacupuncture stimulation for analgesia was appropriate because the amount of cGMP was significantly decreased in rats’ telencephalon and slightly increased in their brainstem during the process, and the pain threshold was significantly enhanced as well. However, Wang [50] found that the overall efficacy of a 45-min acupuncture treatment for KOA was better than that of the 30-min one, using the same position, acupoints, and techniques.

There is a lack of universal clinical guidelines for the frequency of acupuncture treatment [51]. The best efficacy of acupuncture for any disease could only be achieved by the optimal amount of stimulation. The interval of acupuncture often affects the amount of stimulation and thus the efficacy [52]. Our study showed that a frequency of once a day was the most applied frequency of treatment, followed by three times per week and once every other day. Liu et al. [53] studied the modulating effect of electroacupuncture with various intervals treating neuropathic hyperalgesia and found that the effective intervals that could reduce the degree of hyperalgesia were once daily or once every other day, which also promoted the recovery of nerve injury. Moreover, both intervals had a comparable effect on relieving symptoms [53, 54]. Zhao et al. [55] explored the analgesic effect of electroacupuncture in rats and concluded that there was no analgesic effect when the interval was 3 h or 6 h, while 12 h or 24 h interval produced analgesic effect, and the effect was best at 24 h. Wang et al. [56] also found that among different intervals, the 24 h one was more effective in significantly reducing pain level and increasing pain threshold in inflammatory pain rats and promoting POMC mRNA and PENK mRNA expression. Liu et al. [57] compared the analgesic effect of electroacupuncture at different intensities and frequencies in a chronic constrictive injury (CCI) pain model caused by ligating the sciatic nerve in rats. They found that in EA treatment of lower intensity, once-per-day treatment had a better analgesic effect compared with frequencies of once every other day and once a week, which is consistent with our results. Furthermore, the duration of the acupuncture effect is limited. After the dynamic observation of acupuncture effect, Tang et al. [58] proposed that acupuncture had an instant effect that gradually weakened from the peak. Since the instant effect was the basis for the long-term efficacy (after-effect), it was suggested that properly shortening the interval and increasing the times of treatment to strengthen the instant effect might contribute to the improvement of the overall efficacy, and frequency of once per day seemed to be a good choice for treating KOA.

The analysis of channels showed that clinical electroacupuncture treatment for KOA attached importance to point selection along the affected channels. Among the articles included, the Stomach Channel of Foot Yangming had the most acupoints selected. As a channel with sufficient qi and blood, it can nourish all the viscera and bowels, channels, limbs, bones, skins, and tendons when the body is in a healthy state, conducing to strong muscles, flexible joints, and free movement [59]. In *Inner Canon of Yellow Emperor*, the earliest Chinese medical book on basic TCM theories, it says, “Treating flaccidity syndrome by selecting Yangming channel alone.” “Yangming channel is the source of five viscera and six bowels. It can nourish the tendons, which are responsible for constraining the bones and making the joints move freely [60].” Other frequently used channels include the Spleen Channel of Foot Taiyin, the extra points (non-channel points), and the Gallbladder Channel of Foot Shaoyang. All of the above pass through the knee, the main lesion site of KOA, which conforms to the rule that “the channel can treat wherever it passes [61].”

Frequency analysis of acupoints showed that local points were most selected in treating KOA, including Dubi (ST35), Liangqiu (ST34), Neixiyan (EX-LE4), Xuehai (SP10), Yanglingquan (GB34), and Yinlingquan (SP9), which was substantiated in several other studies [61–63]. A literature review by Chen et al. [64] also showed that the top six acupoints with the highest frequency of sensitization in KOA were Yanglingquan (GB34), Xuehai (SP10), Dubi (ST35), Neixiyan (EX-LE4), Yinlingquan (SP9), and Liangqiu (ST34), in descending order. Besides, the most used acupoints included Zusanli (ST36), Ashi point (ouch point), Xiyan (EX-LE5), Heding (EX-LE2), and Xiyangguan (GB33), all of which are located around the knee, in line with the rule that “the acupoint can treat where it is located” [65]. Dubi (ST 35) belongs to the Stomach Channel of Foot Yangming and Neixiyan (EX-LE4) belongs to the extra point. Both can unblock the channels, disperse wind, dissipate cold, free the joints, and relieve pain. Yanglingquan (GB34), and Yinlingquan (SP9) were He-sea points of the Spleen Channel of Foot Taiyin and the Gallbladder Channel of Foot Shaoyang, respectively. They can relax sinews, activate collaterals, dispel wind, eliminate dampness, and remove obstruction when used together. Xuehai (SP10), the cleft of blood, belongs to the Spleen Channel of Foot Taiyin. Since the spleen regulates the blood, needling the point can activate blood, harmonize the nutrient, and unblock the channel [66]. Liangqiu (ST 34), the Xi-cleft point of the Stomach Channel where the channel qi accumulates deeply, helps harmonize qi and blood.

The association analysis of acupoints showed that Liangqiu (ST34) and Xuehai (SP10) had a stronger association, both of which were local points around the knee. Matched as an exterior–interior point combination, they can tonify and activate blood, unblock the channel, and relieve pain. A study by Wang et al. [67] has demonstrated that needling these two points regulated qi and blood, balanced muscle dynamics around the knee, and promoted the recovery of joint function. Cluster analysis indicated two major categories among the acupoints used at least four times. One was three groups of acupoints: Dubi (ST35)–Liangqiu (ST34)–Neixiyan (EX-LE4), Xuehai (SP10)–Yanglingquan (GB34), and Yinlingquan (SP9)–Zusanli (ST36). Zusanli (ST36), the He-sea point of the Stomach Channel of Foot Yangming, which helps regulate the stomach and spleen, tonify qi and blood, unblock the channel, and eliminate the pathogenic factors, is an important acupoint in the treatment of KOA [61, 68]. Along with the frequency analysis and association analysis, we considered this category as the obligatory acupoints. The other category was Ququan (LR8)–Xiyan (EX-LE5)–Xiyangguan (GB33)–Ashi point (Ouch point)–Heding (EX-LE2), which was considered as adjunctive acupoints.

In addition, we have to admit that our study has some limitations. The quality of the included articles was uneven. Patients with syndrome differentiation were not classified for further research, and some of the data unclear in the articles were generally processed, which may have affected the results.

## Conclusions

Continuous wave, low frequency of current (2 Hz), 30-min stimulation, and local acupoint selection are frequently used for electroacupuncture treating knee osteoarthritis. Due to the limitations of this study, further research and more standardized, multi-centered, and large-sample clinical trials could be conducted for more convincing evidence.

## Data Availability

All data generated or analyzed during this study are included in this published article.

## Abbreviations

CNS: Central nervous system
EA: Electroacupuncture
KOA: Knee osteoarthritis
LHS: Left-hand side
RHS: Right-hand side
TCM: Traditional Chinese medicine
